# Presence of Circulating Tumor Cells Predates Imaging Detection of Relapse in Patients with Stage III Melanoma

**DOI:** 10.3390/cancers15143630

**Published:** 2023-07-15

**Authors:** Anthony Lucci, Sridevi Addanki, Yi-Ju Chiang, Salyna Meas, Vanessa N. Sarli, Joshua R. Upshaw, Mayank Manchem, Sapna P. Patel, Jennifer A. Wargo, Jeffrey E. Gershenwald, Merrick I. Ross

**Affiliations:** 1 Departments of Breast Surgical Oncology and Surgical Oncology, The University of Texas MD Anderson Cancer Center, Houston, TX 77030, USA; saddanki@mdanderson.org (S.A.); smeas@mdanderson.org (S.M.); vnsarli@mdanderson.org (V.N.S.); jrupshaw@mdanderson.org (J.R.U.); s2098055@online.houstonisd.org (M.M.); 2 Department of Surgical Oncology, The University of Texas MD Anderson Cancer Center, Houston, TX 77030, USA; ychiang1@mdanderson.org (Y.-J.C.); jwargo@mdanderson.org (J.A.W.); jgershen@mdanderson.org (J.E.G.); mross@mdanderson.org (M.I.R.); 3 Department of Melanoma Medical Oncology, The University of Texas MD Anderson Cancer Center, Houston, TX 77030, USA; sppatel@mdanderson.org

**Keywords:** melanoma, CTC, circulating tumor cell, radiologic surveillance, liquid biopsy, biomarker

## Abstract

**Simple Summary:**

In this study, we investigated how frequently and how early circulating tumor cells (CTCs) were identified prior to the surveillance imaging detection of melanoma progression. This paper reports the results from 325 stage III melanoma patients from a prospective, IRB-approved study at our institution. These patients had blood drawn at baseline and then every 6–12 months to identify CTCs up to 3.5 years from diagnosis. Imaging (CT, PET/CT, MRI, and/or ultrasound) was conducted at baseline and then every 3 months as standard follow-up. We found that CTCs were detected in 76% of stage III melanoma patients who eventually had radiologically detected disease recurrence/metastasis and were identified at a median time of 9 months before imaging confirmation of disease progression. We believe that this finding is important as it provides the basis for future studies using CTCs to risk stratifying melanoma patients.

**Abstract:**

Stage III melanoma includes nodal metastasis or in-transit disease. Five-year survival rates vary between 32% and 93%. The identification of high-risk patients is important for clinical decision making. We demonstrated previously that ≥1 circulating tumor cells (CTCs) at baseline was associated with recurrence. In this study, we investigated how frequently CTCs were identified prior to radiologically detected recurrence. Stage III patients (*n* = 325) had imaging at baseline and q 3 months. Baseline and q 6–12 months blood draws (7.5 mL) were performed to identify CTCs up to 3.5 years from diagnosis. CTC assessment was performed using the immunomagnetic capture of CD146-positive cells and anti-MEL-PE. The presence of one or more CTCs was considered positive. We analyzed the cohort of patients with relapse confirmed by radiologic imaging. CTC collection dates were assessed to determine the lead time for CTC detection. CTC-negative patients were significantly less likely to relapse compared to patients positive for CTCs (*p*-value < 0.001). Within the 325-patient cohort, 143 patients (44%) had recurrence, with a median follow-up of 52 months from diagnosis. The cohort (*n* = 143) with positive imaging and CTC results revealed 76% of patients (108/143) had CTC+ results before the radiological identification of relapse. The median time between positive CTC and positive imaging was 9 months. CTCs were positive in >75% of patients prior to relapse at a median of 9 months before radiologic detection.

## 1. Introduction

Cutaneous melanoma is an aggressive cancer that develops in the skin’s melanocytes, most frequently due to long-term exposure to ultraviolet emissions from the sun [1,2]. There has been an increase in the prevalence of melanoma worldwide, especially in Western countries such as the United States [3]. In 2022, it was estimated that almost 100,000 people were diagnosed with melanoma of the skin in the U.S, and approximately 7650 patients succumbed to the disease [4]. 

The presence of nodal, cutaneous, local/satellitosis, or in-transit metastases characterizes stage III melanoma. Based on T and N scores, the American Joint Committee on Cancer’s (AJCC) eighth edition distinguishes four stage III groups (IIIA, IIIB, IIIC, and IIID) [5,6]. Stage III has 5-year melanoma-specific survival ranging from 93% (IIIA) to 32% (IIID) [4]. Similarly, the risk of disease relapse after surgery is significantly different for these patients. The progression-free and overall survival of patients with metastatic melanoma improved after the introduction of FDA-approved targeted treatments and immune checkpoint inhibitor therapies [7,8,9,10]. However, the efficacies of these drugs are often limited by the development of acquired resistance, leading to disease progression [11,12,13]. Treatment with Ipilimumab, an inhibitor of cytotoxic T-lymphocyte antigen 4, revealed that a subpopulation of patients are long-term survivors, highlighting the need for predictive biomarkers to stratify these patients [9]. Identifying those stage III patients who are at high risk of relapse is important since these immunotherapies have been shown to decrease the risk of relapse. However, these treatments can have significant side effects, so they are used selectively in patients with stage III disease. It would therefore be optimal to use such treatments in the high-risk groups who stand to benefit most, while potentially avoiding their use in lower-risk populations where the possible benefit is smaller. Despite routine surveillance measures that include expensive imaging modalities such as PET/CT scans, disease relapse in patients with stage III melanoma can go undetected or missed in between scans, allowing additional time for disease progression before treatment is initiated. This highlights the need for additional longitudinal monitoring of patients to improve surveillance methods and allow for earlier treatment intervention. Due to the fact that they are minimally invasive compared to the limitations presented by tumor biopsies, liquid biopsies are becoming a more relevant and important approach to this problem.

Circulating tumor cells (CTCs) are cancer cells that are shed into circulation either from a primary or a metastatic tumor site, with the potential for subsequent metastases [14,15]. Circulating tumor markers such as CTCs, circulating tumor DNA (ctDNA), and circulating microRNAs (miRNA) have been reported to be prognostic factors in patients with stage III and IV melanoma [16,17,18]. Due to the propensity of melanoma to spread to distant sites, CTCs have demonstrated a strong prognostic value, as evaluated by many studies [19,20,21]. We earlier reported the potential of CTCs to identify patients likely to suffer disease progression in stage IV melanoma [22]. CTCs may have even higher clinical utility in patients with stage III melanoma because of their varied 5-year survival rates and the ability to intervene with treatments aimed at potential cure. We also found in an earlier study from our group that in stage III melanoma, the presence of one or more CTCs was independently associated with disease relapse [23]. Therefore, it appears that CTCs can potentially serve as an early indicator of melanoma relapse. Also, we hypothesize that since CTC liquid biopsies detect disease at the single-cell(s) stage, they should outperform standard imaging such as CT or PET scans in detecting early recurrence since even the most sensitive imaging modalities require much larger metastasis volumes at their current thresholds of detection to render a positive result. In this study, we tested our hypothesis that CTCs can be detected in patients with stage III melanoma prior to radiologic confirmation of disease progression, and we measured how far in advance of a positive imaging result we could detect the CTCs. We believe that the identification of a blood-based biomarker that is associated with relapse, and that can be identified before standard radiologic imaging, could improve outcomes by allowing for early systemic intervention, especially in those patients not currently on active treatment. The following is a report of our findings.

## 2. Materials and Methods

### 2.1. Patients

Patients with biopsy-proven (positive sentinel node/lymph node(s), satellitosis, clinical regional nodal disease, etc.) AJCC stage IIIA-IIID melanoma were enrolled onto an institutional-review-board-approved protocol (The University of Texas MD Anderson Cancer Center LAB11-0314; Principal Investigator, A. Lucci) between January 2012 and February 2022 to evaluate the detection of circulating tumor cells in peripheral venous blood. Most patients in this study were enrolled at initial stage III diagnosis; a smaller percentage were enrolled at recurrent stage III disease. This study was conducted in accordance with the principles of the Declaration of Helsinki, and informed consent was obtained from all patients prior to initiating any study activities. Clinicopathological factors were collected and included in Table 1. Patient blood samples were drawn within 30 days from the time of study enrollment and/or at follow-ups approximately every 6–12 months for up to three and a half years from study entrance. Baseline was defined as blood draw collected at time of stage III diagnosis (pathologically confirmed by positive sentinel lymph node biopsy, and/or core biopsy of regional lymph node, and/or biopsy of in-transit/satellite lesions). No adverse events or complications from the blood draws were reported during study participation.

### 2.2. Study Design

Patients were pathologically staged according to the AJCC 8th edition for stage III cutaneous melanoma [24]. After initial assessment and intervention, patients proceeded with standard radiologic imaging surveillance occurring approximately every 3–6 months from diagnosis. The imaging modalities used to monitor for disease relapse consisted of PET/CT, CT chest/abdomen/pelvis, MRI (usually to evaluate brain and bone or special areas of concern), or ultrasound (with FNA or core biopsy to further evaluate areas such as lymph nodes or subcutaneous lesions). Relapse was defined as biopsy-proven recurrence, progression, or metastasis of melanoma at the primary tumor site, in transit lesion(s), lymph node involvement, or distant site(s). CTC collection dates and results were compared with follow-up imaging to determine the lead time of relapse should the patient have had a positive CTC result prior to positive imaging and biopsy confirmation. Blood draws were performed at baseline and at each follow-up visit of 6–12-month intervals; since imaging was usually performed at the same follow-up visit, most collections were aligned with imaging. Patients with positive CTC results but negative imaging were also examined to consider the impact on predictive value. The presence of ≥1 CTC was considered a positive result based on previously published studies using CTCs and stage III melanoma [23]. Patients who did not have CTC data available—or a minimum of six months of long-term follow-up—were excluded from the analysis. Follow-up time was defined as the date of sentinel lymph node biopsy or complete lymph node dissection to last vital date or death. In cases where patients did not undergo surgical intervention, date of no evidence of disease (NED) confirmation was used as the start time. 

### 2.3. Isolation and Enumeration of CTCs

All samples were processed in the same lab at MD Anderson, Houston, TX. The Celltracks® Circulating Melanoma Cell Kit (Menarini Silicon Biosystems) was used to analyze CTCs [25]. CellSearch is an immuno-based method for detecting and isolating CTCs from blood and has received FDA approval. The identification of CTCs by immunomagnetic enrichment is conventionally performed by positive selection for epithelial cell-surface markers such as EpCAM and cytokeratins. Melanoma cells originate from neural crest cells and thus do not express EpCAM. Instead, melanoma cells often express the cell adhesion molecule CD146, which has been shown to be a useful marker for CTC capture in liquid biopsy for melanoma [26]. The circulating melanoma cell assay involves the enrichment of CTCs from the whole blood by binding the cells to the anti-CD146-antibody-conjugated iron nanoparticles, followed by magnetic capture. Circulating melanoma cells are further identified using fluorescently labeled antibodies to detect a combination of high-molecular-weight melanoma-associated antigen (HMW-MAA; clone 9.2.27, Janssen, LLC), CD45, and CD34. Anti-CD45 staining will allow for the differentiation of CTCs from circulating WBCs. Thus, circulating melanoma cells were defined as CD146+, HMW-MAA+, CD45−, CD34−, and nucleated (DAPI+) cells.

A 10 mL Cell Save tube with a proprietary preservative was used to collect a minimum of 7.5 mL of peripheral venous blood. Following manufacturer’s instructions, samples were processed for CTC evaluation within 96 h of blood collection. For the immunomagnetic enrichment of melanoma cells, ferrofluids coated with CD146 antibodies were used along with fluorescently labeled melanoma-specific antibody (HMW-MAA) for CTC detection, anti-CD45 as a negative control to exclude leukocytes, and anti-CD34 for endothelial cells. All results were read by three highly experienced technicians with a combined 14 years of experience reading CTC results on CellSearch. All interpreters were blinded to clinical data.

### 2.4. Statistical Analyses

The difference in demographic and clinicopathological features between CTC presence (positive/negative) in relapse patients was tested using the Mann–Whitney U-test for continuous data, and Chi-square test or Fisher exact test for categorical data. Univariate logistic regression analysis was applied to determine factors (i.e., CTC, age, gender, race, primary tumor ulceration, etc.) for predicting relapse of the whole cohort and predicting CTC presence (positive/negative) within patients who relapsed. The Kaplan–Meier method was used to calculate the survival curves, and the log-rank test was applied to compare the curves of CTC presence (positive/negative) of the overall cohort and within the relapse group. The counting process method was applied in COX proportional hazards regression analysis to estimate hazard ratio (HR) and 95% confidence intervals (CI) for factors associated with the risk of relapse-free survival. Three multivariate regression models were performed, time-dependent COX model, Cox model, and logistic regression model, to assess multiple statistical outcomes, avoid potential bias, and so that time to CTC > 1 detection and time to positive imaging could be considered time-dependent covariates [27]. Univariate Cox proportional hazards regression analysis was used to estimate the hazard ratio (HR) and 95% confidence intervals (CI) for the factors associated with risk of relapse-free survival (RFS). Time-dependent receiver operating characteristic (ROC) curve analysis was conducted to evaluate the area under the curve (AUC) of the multivariate regression model. Sensitivity and specificity were also calculated. Statistical analysis was performed using the SAS Enterprise Guide 7.15 (SAS Inc., Cary, NC, USA). All tests were 2-sided. Statistical significance was defined as *p* < 0.05.

## 3. Results

### 3.1. Patient Characteristics

A total of 325 patients with stage III melanoma were enrolled in this study. Patient characteristics are reported in Table 1. The overall study cohort was comprised of 174 males (53.5%) and 151 females (46.5%). The mean age was 54.3 years (range, 20–89 years). The median follow-up time at stage III diagnosis was 48.3 months (range, 7.1–262.3 months). In total, 96% percent of study patients were white, while 4% were of other races. Out of 325 (22%) patients, 70 had pathologic stage IIIA disease, 82 (25%) were stage IIIB, 155 (48%) were stage IIIC, 14 (4%) were stage IIID, and 4 (1%) were stage III unclassified. The most common histologic types were superficial spreading (121/325, 37%) and nodular (62/325, 19%). One hundred and forty-three (44%) patients experienced disease relapse during the study period. Relapse consisted of 6% local (9/143), 17% in transit (24/143), 31% regional node (44/143), and 46% distant metastasis (66/143). Of the 143 patients who relapsed, 5% were stage IIIA (7/143), 20% were stage IIIB (29/143), 69% were stage IIIC (98/143), and 6% were stage IIID (8/143). This cohort consisted of 42% females (60/143) and 58% males (83/143) with a median age of 58 years (range 27–83). One hundred and eighty-two (56%) patients did not experience disease relapse.

### 3.2. Presence of CTCs Prior to Positive Imaging

The subset of patients (*n* = 143) with positive imaging and CTC data was evaluated to determine how often—and how far ahead temporally—that CTC identification predated positive imaging findings. The patient characteristics for this subset are reported in Table 1. Out of 143, 108 patients (76%) had a positive CTC result detected prior to the radiographical identification of relapse. Out of 108 patients, 21% were stage IIIB (23/108), while 70% were stage IIIC (76/108). Patients who relapsed with a positive CTC result predating imaging had the following types of relapse event: local (3%), in transit (19%), regional node (33%), and distant metastasis (44%). CTCs predated imaging modalities that included CT (42%), PET/CT (24%), MRI (17%), and ultrasound (23%). The rates of the identification of metastasis were not significantly different between CT, PET, and PET/CT. The median number of CTC samples collected per patient was two (range 1–5) blood draws prior to positive imaging. Out of the 108 patients who had CTCs identified prior to positive imaging, 73 completed more than one CTC blood draw. The median time between each draw was approximately 7 months. The range between each sample collection varied (draw 1–2: 2 weeks-37 months, draw 2–3: 1–24 months, draw 3–4: 4–23 months, draw 4–5: 3–12 months). All 108 CTC-positive patients had at least one CTC detected. Most patients had one CTC detected prior to positive imaging (64/108, 59%), while 32 had between two and three CTCs detected (30%), and 12 had more than three (11%) CTCs per 7.5 mL tubes of blood prior to positive imaging. The median number of CTCs detected was one CTC. Most patients had CTCs first detected at their first blood draw (66/108, 61%), while 25 patients had CTCs first detected at draw 2, 15 patients at draw 3, 1 patient at draw 4, and 1 patient at draw 5. The median time between positive CTC result and positive imaging was 9 months. In total, 70 patients (70/108, 65%) were observation only, while 38 (38/108, 35%) were on systemic treatment prior to relapse.

### 3.3. CTCs Detected in Patients without Relapse

Out of 182 patients who did not experience disease relapse, 109 (60%) patients had a positive CTC detected at least one time throughout surveillance despite negative imaging (Table 2). The median follow-up time for these patients was 47.7 months. Out of the 109 patients with a positive CTC detected, 13 did not complete a follow-up blood draw, while 96 patients had two or more blood draws to evaluate. In this group (*n* = 96), 53% of patients (51/96) were observed to be on systemic treatment (immunotherapy or targeted therapy) after a positive CTC was detected. This includes patients who had a blood draw before, during, or at any interval after systemic treatment. Of the 51 patients who were CTC-positive and on systemic treatment, 25 (49%) patients achieved CTC clearance, with an eventual result of zero CTCs detected, throughout treatment. It was also observed that 21 (21/45, 47%) patients who were CTC-positive and not on systemic treatment also achieved CTC clearance at subsequent draw(s). 

### 3.4. Absence of CTCs Prior to Positive Imaging

Out of 143 patients with positive imaging and CTC data, 35 patients (24%) had no CTCs detected prior to positive imaging (Table 1). The median time between earliest CTC sample collection and relapse confirmed by imaging was 13.1 months. The median number of CTC sample collection per patient was two (range 1–3) blood draws prior to positive imaging. Most patients were stage III B (17%) and III C (63%). Patients who relapsed with negative CTC result(s) predating imaging had the following types of relapse events: local (17%), in transit (9%), regional (23%), and distant metastasis (51%). Imaging modalities included CT (49%), PET/CT (23%), MRI (14%), and ultrasound (14%). Ultrasound was commonly used as an adjunct to systemic staging studies, usually to confirm and/or biopsy regional nodal disease or subcutaneous nodules. In total, 22 patients (63%) were observation only, while 13 patients (37%) were on systemic treatment prior to relapse.

### 3.5. Comparison of CTC-Positive and CTC-Negative Groups Prior to Positive Imaging

**Figure 1:**
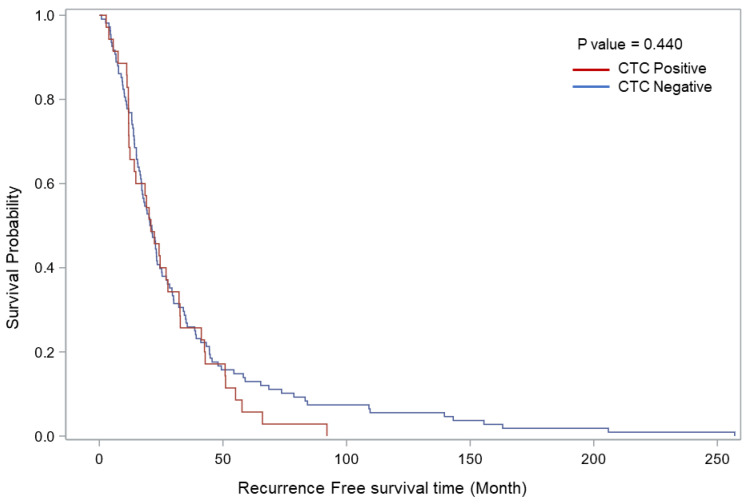
Kaplan–Meier relapse-free survival curves comparing patients with stage III melanoma with and without CTCs detected prior to positive imaging. The figure shows the probability of relapse-free survival in patients with or without CTC detected prior to positive imaging (log rank *p* < 0.440).

Table 1 also shows there were no significant associations between demographic/clinicopathological features and CTC presence in patients with confirmed relapse (pathologically in almost all cases except MRI-detected brain lesions). Patients with CTCs detected prior to positive imaging had more blood draws collected than patients with zero CTCs detected (Table 1). Univariate logistic regression analysis (Table 3) showed no significant association between covariates and CTC presence as an outcome in the relapse group. The AUC of the multivariate regression model showed a value of 73%, indicating an association with CTCs and relapse. The sensitivity and specificity of the CTC test was 76% and 40%, respectively. The median recurrence time from the first positive blood draw was 9 months in patients with CTCs detected prior to positive imaging, which was significantly shorter than patients with no CTCs detected prior to positive imaging (median recurrence time was 13.1 months). In Figure 1, Kaplan–Meier survival analysis did not reach significance for patients with CTCs detected prior to positive imaging compared to patients with no CTCs detected prior to imaging, though the Kaplan–Meier analysis demonstrated significantly worse disease-free survival for the overall cohort (see below). 

### 3.6. Correlation of CTC Presence and Relapse in Overall Cohort

**Figure 2:**
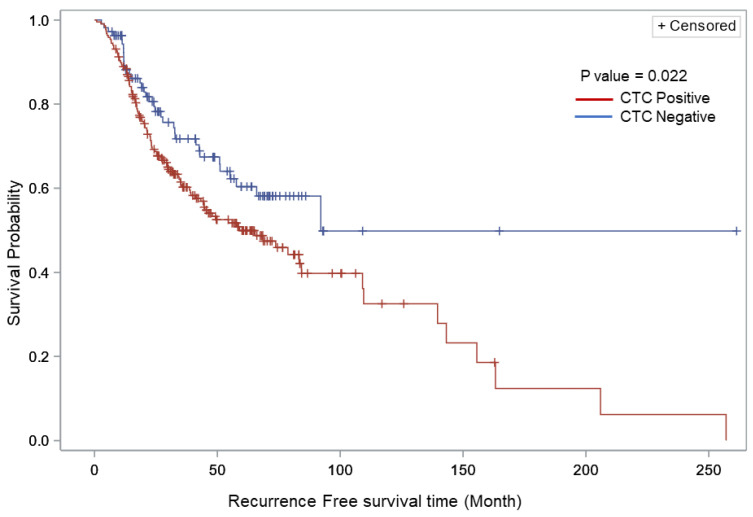
Kaplan–Meier relapse-free survival curves comparing CTC-positive vs. CTC-negative patients with stage III melanoma within the overall cohort. The figure shows the probability of relapse-free survival in patients with or without CTC detected at any time (log rank *p* < 0.022).

Univariate logistic regression analysis of the overall cohort (Table 4) was performed to assess the correlation between covariates and relapse within the overall cohort (*n* = 325). Patients who were CTC-negative were significantly less likely to develop disease relapse compared to those who were CTC-positive (OR: 0.48, 95% CI: 0.30–0.78, *p*-value < 0.006). The presence of at least 1 CTC was associated with significantly increased risk of disease relapse (OR: 2.07, 95% CI: 1.28–3.35, *p*-value < 0.003). Kaplan–Meier survival analysis showed that the presence of CTCs was significantly associated with decreased relapse-free survival (*p* = 0.022, log-rank test, Figure 2). Univariate cox regression analysis (Table 5) was performed to evaluate time to recurrence for CTC presence (positive/negative) in the overall cohort. The analysis showed that CTC-negative patients had a lower risk of relapse than CTC-positive patients (HR: 0.37, 95% CI: 0.26–0.53, *p*-value < 0.001). In Table 6, multivariate cox regression analysis using CTC as a time-dependent covariate showed that patients with a higher number of lymph nodes containing metastases had increased risk of relapse compared to patients with one positive lymph node, and the hazard ratios increased along with the number of nodes (HR: 1.64 and 2.68 of 2–3 LN and >3 LN, *p* < 0.001). Similarly, the hazard ratios for relapse increased concomitantly with the number of CTCs detected (*p* < 0.001).

### 3.7. Representative Case Examples

**Figure 3:**
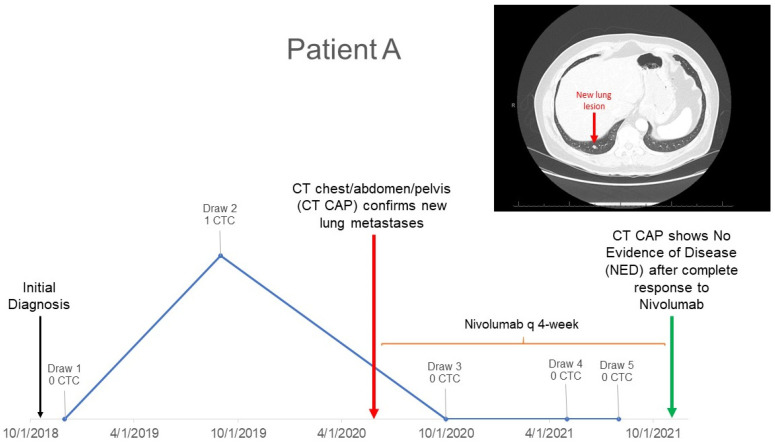
Patient A with positive CTC detected prior to positive imaging who achieves CTC clearance.

**Figure 4:**
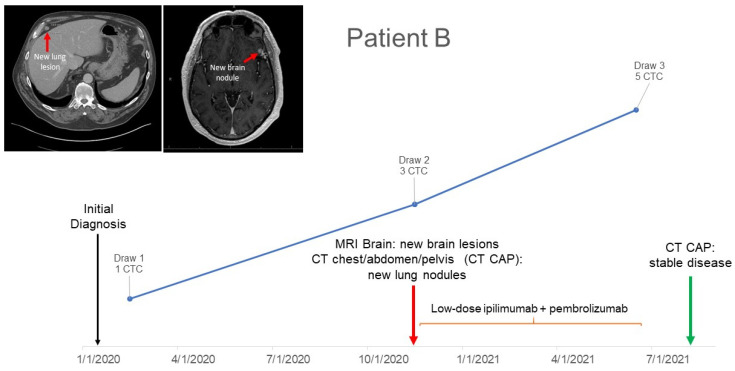
Patient B with positive CTC detected prior to positive imaging with subsequent CTC detection throughout prognosis.

In our current study, we demonstrated that CTCs could, in many cases, be identified many months prior to the confirmed radiologic detection of disease relapse. To further highlight this finding, we provide example cases where CTCs detected months prior to positive imaging were shown to correlate with prognostic outcomes. In Figure 3, we show Patient A who had one CTC detected approximately 8 months prior to CT scans of chest/abdomen/pelvis showing lung metastases. Patient A proceeds to achieve CTC clearance, as indicated by their subsequent results of zero CTCs. This CTC clearance was confirmed to correspond with the patient’s clinical outcome—no evidence of disease (NED) due to complete response to immunotherapy. Figure 4 illustrates Patient B’s CTC results which begin with a positive CTC result of one approximately 8 months prior to the MRI detection of a new brain lesion and CT detection of new pulmonary nodules. Subsequent blood draws for Patient B resulted in increasing numbers of CTCs, which corresponded with further progression and poor prognosis. While these are only select examples, they highlight the potential future clinical utility for early intervention or changes in systemic therapy to attempt to head off the establishment of distant metastasis.

## 4. Discussion

Even though routine follow-up staging studies—including CT, PET/CT, or PET scans—have traditionally been used to monitor disease response and recurrence in high-risk patients with melanoma, the benefits can be hindered by technical limitations, cost-effectiveness, and patient discomfort. In clinical practice, a liquid biopsy that can detect relapse at an earlier stage, before a clinical metastasis is formed, could be useful to more effectively guide the timing of imaging, or to allow for early intervention with effective treatments before clinically detectable metastases are established. Our results presented herein showed that CTCs were identified in over 3/4ths of patients prior to the imaging identification of relapse. Furthermore, the CTCs were found at a median of over 9 months before the radiological detection of progression, meaning that there is significant lead time available for treatment intervention. Finally, the presence of CTCs was significantly associated with melanoma relapse, suggesting that circulating tumor cells should be investigated further as a possible blood-based biomarker to identify those patients at high risk of relapse, and allow for the initiation of systemic treatment, especially in those patients who are only on observation/no active treatment. The detection of CTCs prior to radiological confirmation provides an opportunity for earlier clinical intervention, which may lead to increased overall survival and relapse-free survival for patients with stage III melanoma. In our previous study, we used single baseline measurements and found that CTCs had the same associations with relapse. In this current study, we use serial CTC measurements and compared CTC positivity to the eventual documentation of disease progression on routine imaging. Both studies confirm a possible role for CTCs in detecting patients at higher risk of relapse. In current clinical practice, there are no blood biomarkers to guide oncologists as to which patients with melanoma are at highest risk of relapse, and which patients can avoid systemic therapy since they are at low risk. The development of an effective blood biomarker would therefore be of significant clinical utility in clinical melanoma management. 

Liquid biopsy measurements using CTCs have several advantages over radiological findings. Blood draws to assess CTCs can often be scheduled at the time of regular follow-up labs, avoiding multiple sticks and maximizing patient comfort. Staging studies are also far more expensive (average CT and PET scan cost: USD 7000–11,000) than routine CTC assessment (average cost in our lab USD 375.00); therefore, serial CTC biomarker studies would be highly cost-effective compared to repeating CTs or PET/CTs every three months, resulting in a significant reduction in healthcare costs. Most importantly, our results showed that there was lead time of over 9 months when using CTC assessment compared to imaging studies, which is an important finding clinically as it allows for significant lead time to begin systemic treatments and possibly head off metastatic disease development. Further study is needed to assess if systemic therapies can eradicate CTCs and prevent metastasis. This pilot study was not designed to determine if immunotherapies or targeted therapies could eradicate CTCs. However, future studies designed with giving immunotherapy in a neoadjuvant fashion (for example, stage III diagnosis and neoadjuvant systemic therapy followed by surgical clearance of residual disease), with blood draws before, during, and after treatment, will be conducted to study that exact issue in a more controlled manner.

The finding that most patients who eventually had relapse identified on their imaging also had a positive CTC result was not especially surprising. We had previously conducted a feasibility trial to see if we could identify CTCs at baseline in a group of 93 patients with stage IV melanoma, and, if so, did CTCs have prognostic significance? [22]. In that study, we found that 42% of stage IV patients had a positive baseline CTC result, and that the presence of CTCs was significantly associated with disease progression. Out of 93 (30%) patients in that study, 28 progressed within 180 days of baseline draw, with 20 of 39 (51%) of the CTC-positive patients relapsing compared with 8 of 54 (15%) of the CTC-negative patients. Similar results were seen in our first evaluation of baseline CTC results in 243 patients with stage III cutaneous melanoma in different sub-stages [23]. That study revealed that 37% of baseline patient blood samples contained CTCs, and that 14% of those patients developed a disease relapse within six months of the baseline CTC detection. That study identified CTCs in one-third of the entire cohort of patients with stage III melanoma, while in this study, we focused on those who eventually developed relapse—the group that one would hypothesize would be most likely to be shedding CTCs. Indeed, the finding that 76% of the patients in this study had at least one positive CTC result supports that hypothesis. Finally, it appears that multiple blood draws improve the rates of CTC detection as, in some cases, patients may have a negative result, followed by a positive result, and vice versa. Serial draws improve yield and paint a dynamic picture of the disease response/progression process. Thus, future studies should incorporate serial blood draws at the defined follow-up points and at time of relapse.

The idea of comparing CTC assessment to imaging has been reported previously, mostly in other disease sites. A study by Budd et al. showed that patients with metastatic breast cancer who had >5 CTCs in 7.5 mL of blood before and/or after receiving systemic therapy had shorter progression-free and overall survival times [28]. They compared CTCs and radiographic findings 10 weeks after systemic treatment and found that median overall survival of 13 (9%) patients with radiologic non-progression and ≥ 5 CTCs was significantly shorter than that of the 83 (60%) patients with radiologic non-progression and <5 CTCs (15.3 vs. 26.9 months; *p* = 0.0389). They concluded that CTCs identification was an earlier and more reproducible assessment of disease status compared to current imaging. They also concluded that CTCs may be a superior surrogate end point in metastatic breast cancer, noting that they were highly reproducible and correlated better with survival than did changes found on traditional imaging studies.

Regarding melanoma, Koyanagi et al. performed a study where blood samples were taken from 63 patients with stage III melanoma receiving neoadjuvant biochemotherapy before and after surgical treatment [29]. The study’s objective was to evaluate the efficacy of CTCs (in this case, assessed by quantitative real-time reverse transcriptase polymerase chain reaction for the expression of four melanoma-associated markers) as a surrogate marker of outcomes in patients with stage III melanoma by predicting tumor progression and therapy response. Their study found that marker detection post-treatment was associated with significant decreases in relapse-free and overall survival. Their results supported the idea that serial blood monitoring for CTCs may help identify residual systemic disease and help clinicians to better assess patient’s risk of relapse. One issue with their study is they used quantitative real-time reverse transcriptase polymerase chain reaction methodology, which is not standardized, and thus would be hard to incorporate into different centers quickly. 

Our results also included a subset of patients who were CTC-negative despite experiencing disease relapse, as well as patients who were CTC-positive but remained with no evidence of disease (NED). This illustrates one of the limitations to our study—it was not designed as a prospective trial with controlled systemic treatment regimens. Thus, we were not able to control for confounders such as treatment effect that might influence CTC clearance, as occurred in some participants on their future blood draws. A recent study by Radovich et al. investigated the association between ctDNA and CTCs after NAC in TNBC patients with disease recurrence [30]. They also observed >50% ctDNA detection in patients who did not disease relapse. A little more than half (56%) of the patients in our study did not experience disease relapse, and this figure could certainly be affected by the use of adjuvant treatments. Of the 109 patients with at least one positive CTC detection event and at least two blood draws and no documented disease relapse, 49% achieved CTC clearance while on treatment (Table 2). With the use of adjuvant treatments, it is possible that CTCs could be eradicated by the host immune system, but this theory needs to be proven in a prospective study.

These results could also be partly attributed to the follow-up period being shorter than sixty months as there could certainly be later relapses with longer follow-up of this group. One must also consider the possibility that current CTC identification technology might miss some positive cells/events. In any case, almost 60% of our patients who had CTCs had only one CTC detected, yet the comparison between the identification of a single CTC (compared to zero CTCs) was highly significantly associated with relapse, supporting the sensitivity of the assay. 

In the future, we plan to design studies with defined CTC serial measurements throughout the systemic treatment course. Currently, CTCs in melanoma are used for research purposes only and not for clinical decision making. However, the data from this study highlight the need for biomarkers associated with melanoma progression, as over 60% of the patients who had a positive CTC result and eventually relapsed were observation only. In the future, the identification of this at-risk group might lead to early intervention with systemic therapy. A controlled prospective study including neoadjuvant and adjuvant systemic treatment groups will provide additional insight on the ability of these agents to eradicate CTCs and improve outcomes. The neoadjuvant approach is highly attractive, especially after the recent report from The European Society of Medical Oncology in Paris in September 2022 showing that a protocol using neoadjuvant pembrolizumab demonstrated significantly prolonged event-free survival compared to the adjuvant arm in the SWOG 1801 trial (HR 0.58, 0.39 TO 0.87; *p* = 0.004) [31]. With a new focus on neoadjuvant immunotherapy for Stage III melanoma, there are opportunities to design companion studies to evaluate blood-based biomarkers to improve disease response assessment and the identification of patients at high risk of relapse based on the presence of microscopic disease after the completion of their neoadjuvant treatment.

Finally, another limitation to our approach may have been limiting our liquid biopsy platform to CTCs assessment alone. The sensitivity of the CTC test (76% in this study) can likely be improved with the addition of other biomarkers. Circulating tumor DNA, or ctDNA, is emerging as a non-invasive liquid biopsy biomarker to measure tumor load and monitor cancer genomes across various malignancies [32]. In a comprehensive analysis of serial ctDNA and functional imaging in patients with metastatic melanoma receiving systemic therapy, ctDNA allowed for the real-time monitoring of tumor burden and genomic changes throughout treatment [33]. The findings also highlight ctDNA analysis as a complementary approach for functional imaging. Similarly, it was demonstrated that ctDNA measurements at surgery enhanced the prediction of metastasis, recurrence, and death in early breast cancer patients receiving neoadjuvant chemotherapy (NACT) [34]. In addition, they found a significant correlation between the levels of ctDNA and the functional tumor volume as determined by magnetic resonance imaging. In metastatic lung cancer, ctDNA detected recurrence earlier than CT scans by six months [35]. Prospective clinical trials evaluating the sensitivity and early event prediction of both ctDNA and CTCs are warranted. It is also possible that by adding ctDNA to CTC assessment, we might strengthen the predictive value. In this regard, however, the translatability of CTC detection as a diagnostic tool in clinical practice will be easier as it already has an FDA-approved, standardized methodology [36].

## 5. Conclusions

This report demonstrated that CTCs were significantly associated with worse outcomes in a group of 325 patients with stage III melanoma. CTCs were also identified in 76% of stage III cases who ultimately developed a relapse that was identified by standard follow-up imaging. Importantly, positive CTC results were found at a median of over 9 months before imaging detected disease progression. This long lead time is important since it opens up the possibility of intervening with new systemic therapy, or changing therapies, in order to potentially block metastasis at the microscopic stage. The use of blood-based biomarkers associated with relapse allows the identification of patients at high risk of relapse and thus early systemic treatment intervention. Prospective clinical trials could then be completed testing early systemic intervention to determine if it will lead to improved survival.

## Figures and Tables

**Table 1:** Patient characteristics.

Variables	CTC Predating Positive Imaging			
	Overall Cohort *n* = 325 (%)	Positive CTC *n* = 108 (%)	Negative CTC *n* = 35 (%)	*p*-Value
Total participants	325	108	35	
Age, years, mean (range)	54.3 (20–89)	58.5 (49–67)	55 (28–74)	0.078
Gender				0.901
Male	174 (53.5)	63 (58.3)	20 (57.1)	
Female	151 (46.5)	45 (41.7)	15 (42.9)	
Race				0.999
White	312 (96.0)	103 (95.4)	34 (97.1)	
Other	13 (4.0)	5 (4.6)	1 (2.9)	
Median follow-up, months (range)	48.3 (7.1–262.3)	48.4 (10.6–262.3)	54.8 (9.1–128.6)	
25% follow-up (months)	27.6	26.7	30.4	
75% follow-up (months)	71.3	76	74.5	
Stage III stage group (AJCC 8th Edition)				
III A	70 (21.5)	3 (2.8)	4 (11.4)	
III B	82 (25.2)	23 (21.3)	6 (17.1)	
III C	155 (47.7)	76 (70.4)	22 (62.9)	
III D	14 (4.3)	5 (4.6)	3 (8.6)	
III	4 (1.2)	1 (0.9)	0 (0)	
Vital status				0.408
Alive	265 (81.5)	70 (64.8)	26 (74.3)	
Dead	60 (18.5)	38 (35.2)	9 (25.7)	
Relapse (any recurrence)				
Local	9 (2.8)	3 (2.8)	6 (17.1)	
In transit	24 (7.4)	21 (19.4)	3 (8.6)	
Regional	44 (13.5)	36 (33.3)	8 (22.9)	
Distant	66 (20.3)	48 (44.4)	18 (51.4)	
Histologic subtype				
Superficial spreading	121 (37.2)	32 (29.6)	9 (25.7)	
Nodular	62 (19.1)	17 (15.7)	7 (20.0)	
Acral lentiginous	31 (9.5)	18 (16.7)	4 (11.4)	
Lentigo maligna	10 (3.1)	3 (2.8)	2 (5.7)	
Desmoplastic/mucosal/other	68 (20.9)	25 (23.2)	9 (25.7)	
Missing	33 (10.2)	13 (12.0)	4 (11.4)	
Breslow thickness (mm), median (range)	2.4 (0–45)	3.3 (0–45)	2.2 (0–10)	0.165
Ulceration				
Present	95 (29.2)	25 (23.2)	11 (31.4)	
Absent	108 (33.2)	50 (46.3)	14 (40.0)	
Missing	122 (37.5)	30 (30.6)	10 (28.6)	
Mitotic figures				
<1/mm^2^	9 (2.8)	3 (2.8)	1 (2.9)	
≥1	255 (78.5)	82 (75.9)	26 (74.3)	
Missing	61 (18.8)	23 (21.3)	8 (22.9)	
Tumor-infiltrating lymphocytes				
Absent	1 (0.3)	1 (0.9)	0 (0)	
Brisk	9 (2.8)	3 (2.8)	0 (0)	
Non-brisk	239 (73.5)	75 (69.4)	23 (65.7)	
Missing	76 (23.4)	29 (26.9)	12 (34.3)	
Microscopic satellitosis				
Present	50 (15.4)	27 (25)	6 (17.1)	
Not identified	201 (61.9)	55 (50.9)	19 (54.3)	
Not ruled out	6 (1.9)	3 (2.8)	0 (0)	
Missing	68 (20.8)	23 (21.3)	10 (28.6)	
Total number of regional nodes with metastases				
0 Lymph nodes (LN)	13 (4)	7 (6.5)	4 (11.43)	
1 LN	162 (49.9)	44 (40.7)	8 (22.9)	
2–3 LN	90 (27.7)	32 (29.6)	12 (34.3)	
>3 LN	36 (11.1)	17 (15.7)	9 (25.7)	
Missing	24 (7.4)	8 (7.4)	2 (8.7)	
Distant metastasis				
Present	0 (0)	0 (0)	0 (0)	
Absent	325 (100)	108 (100)	35 (100)	
Imaging modality				
CT		42 (38.9)	17 (48.6)	
PET/CT		24 (22.2)	8 (22.9)	
MRI		17 (15.7)	5 (14.3)	
Ultrasound		23 (21.3)	5 (14.3)	
CTCs detected (any draw)				
0 CTCs	108 (33.2)	0 (0)		
1 CTCs	120 (36.9)	64 (59.3)		
2–3 CTCs	73 (22.5)	32 (29.6)		
>3 CTCs	24 (7.4)	12 (11.1)		
Number of CTC blood draws				
1	92 (28.3)	35 (32.4)	18 (51.4)	
2	89 (27.4)	21 (19.4)	9 (25.7)	
3	116 (35.7)	39 (36.1)	8 (22.9)	
≥4	28 (8.6)	13 (12)	0 (0)	
Treatment status prior to relapse				
Observation only		70 (64.8)	22 (62.9)	
On systemic treatment		38 (35.2)	13 (37.1)	

**Table 2:** Patient characteristics for non-relapsers.

Variables	N = 182 (%)
Age, years, mean (range)	52 (20–89)
Gender	
Male	91 (50)
Female	91 (50)
Race	
White	175 (96.2)
Other	7 (3.8)
Median follow-up, months (range)	47.7 (7.1–261.3)
25% follow-up (months)	26.1
75% follow-up (months)	68.7
Stage III substage (AJCC 8th Edition)	
III A	63 (34.6)
III B	53 (29.1)
III C	57 (31.3)
III D	6 (3.3)
III	3 (1.7)
Vital status	
Alive	169 (92.9)
Dead	13 (7.1)
Histologic subtype	
Superficial spreading	80 (43.9)
Nodular	38 (20.9)
Acral lentiginous	9 (4.9)
Lentigo maligna	5 (2.8)
Desmoplastic/mucosal/other	34 (18.7)
Missing	16 (8.8)
Breslow thickness (mm), median (range)	2 (0.3–19)
Ulceration	
Absent	59 (32.4)
Present	44 (24.2)
Missing	79 (43.4)
Total number of nodes with metastases	
0 lymph nodes (LN)	2 (1.1)
1 LN	110 (60.4)
2–3 LN	46 (25.3)
>3 LN	10 (5.5)
Missing	14 (7.7)
CTCs detected	
0 CTCs	73 (40.1)
≥1 CTCs	109 (59.9)
≥2 CTCs	53 (29.1)
≥3 CTCs	25 (13.7)
CTCs detected	
0 CTCs	73 (40.1)
1 CTCs	56 (30.8)
2–3 CTCs	41 (22.5)
>3 CTCs	12 (6.6)
Number of CTC blood draws	
1	13/109 (11.9)
2	34/109 (31.2)
3	51/109 (46.8)
≥4	11/109 (10.1)
Systemic treatment after CTC-positive (≥2 blood draws, *n* = 96)	
Yes	51/96 (53.1)
No	45/96 (46.9)
Achieved CTC clearance on systemic treatment (*n* = 51)	
Yes	25/51 (49)
No	25/51 (49)
No follow-up draws after CTC-positive	1/51 (2)
Achieved CTC clearance on observation only (*n* = 45)	
Yes	21/45 (46.7)
No	21/45 (46.7)
No follow-up draws after CTC-positive	3/45 (6.7)

**Table 3:** Univariate logistic regression analysis of CTCs detected prior to positive imaging in patients who relapsed (N = 143).

Variables	Odds Ratio	95% Confidence Interval	*p*-Value
Age, years	1.03	1.00–1.06	0.049
Gender			0.901
Male	1.05	0.49–2.27	
Female	reference		
Race			0.653
White	reference		
Other	1.65	0.19–14.63	
Histologic subtype			0.902
Superficial spreading	reference		
Nodular	0.68	0.22–2.16	
Acral lentiginous	1.27	0.34–4.69	
Lentigo maligna	0.42	0.06–2.92	
Desmoplastic/mucosal/other	0.78	0.27–2.26	
Missing	0.91	0.24–3.50	
Breslow thickness (mm) (N = 118)	1.11	0.94–1.29	0.209
Ulceration			0.612
Absent	reference		
Present	0.64	0.25–1.60	
Missing	0.92	0.37–2.33	
Mitotic figures (N = 289)			0.966
<1/mm^2^	reference		
≥1	1.05	0.11–10.55	
Total number of regional nodes with metastases (N = 132)			0.265
1 LN	reference		
2–3 LN	0.49	0.18–1.32	
>3 LN	0.34	0.11–1.04	
Missing	0.73	0.13–4.07	

**Table 4:** Univariate logistic regression analysis of relapse in overall cohort (N = 325).

Variables	Odds Ratio	95% Confidence Interval	*p*-Value
CTC			0.006
Positive	reference		
Negative	0.48	0.30–0.78	
CTCs detected			
≥1 CTCs vs. 0 CTCs	2.07	1.28–3.35	0.003
≥2 CTCs vs. <2 CTCs	1.08	0.67–1.75	0.747
≥3 CTCs vs. <3 CTCs	1.14	0.61–2.12	0.675
CTCs detected			0.016
0 CTCs	reference		
1 CTCs	2.38	1.39–4.09	
2–3 CTCs	1.63	0.88–3.01	
>3 CTCs	2.09	0.85–5.11	
Age, years	1.02	1.01–1.04	0.002
Gender			0.149
Male	1.38	0.89–2.15	
Female	reference		
Race			0.873
White	reference		
Other	1.10	0.36–3.33	
Histologic subtype			0.008
Superficial spreading	reference		
Nodular	1.23	0.65–2.33	
Acral lentiginous	4.77	2.01–11.29	
Lentigo maligna	1.95	0.53–7.13	
Desmoplastic/mucosal/other	1.95	1.06–3.58	
Missing	2.07	0.95–4.52	
Breslow thickness (mm) (N = 294)	1.19	1.08–1.32	<0.001
Ulceration			<0.001
Absent	reference		
Present	0.42	0.24–0.74	
Missing	0.37	0.22–0.64	
Mitotic figures (N = 289)			0.900
<1/mm^2^	reference		
≥1	0.92	0.24–3.49	
Total number of regional nodes with metastases (N = 312)			<0.001
1 LN	reference		
2–3 LN	2.02	1.19–3.43	
>3 LN	5.5	2.47–12.24	
Missing	1.51	0.63–3.63	

**Table 5:** Univariate cox regression analysis of relapse-free survival associated with CTC detection in overall cohort (N = 325).

Variables	Hazard Ratio	95% Confidence Interval	*p*-Value
Age, years	1.02	1.01–1.03	0.001
CTC			<0.001
Positive	reference		
Negative	0.37	0.26–0.53	
Gender			0.009
Male	1.44	1.09–1.89	
Female	reference		
Race			0.83
White	reference		
Other	1.08	0.55–2.10	
Histologic subtype			0.037
Superficial spreading	reference		
Nodular	1.17	0.79–1.72	
Acral lentiginous	2.07	1.34–3.19	
Lentigo maligna	1.01	0.44–2.34	
Desmoplastic/mucosal/other	1.32	0.91–1.91	
Missing	1.42	0.89–2.26	
Breslow thickness (mm), N = 294	1.06	1.03–1.09	<0.001
Ulceration			<0.001
Absent	reference		
Present	0.5	0.36–0.69	
Missing	0.59	0.44–0.81	
Mitotic figures (N = 289)			0.545
<1/mm^2^	reference		
≥1	0.79	0.37–1.69	
Total number of regional nodes with metastases			<0.001
1 LN	reference		
2–3 LN	1.41	1.03–1.95	
>3 LN	2.56	1.73–3.82	
Missing	0.81	0.45–1.48	
CTCs detected			
≥1 CTCs vs. 0 CTCs	2.71	1.89–3.89	<0.001
≥2 CTCs vs. <2 CTCs	1.12	0.85–1.49	0.418
≥3 CTCs vs. <3 CTCs	1.26	0.89–1.80	0.196
CTCs detected			<0.001
0 CTCs	reference		
1 CTCs	3.067	2.10–4.49	
2–3 CTCs	2.26	1.48–3.45	
>3 CTCs	2.44	1.38–4.34	

**Table 6:** Multivariate cox regression analysis of relapse-free survival associated with CTC detection in overall cohort (N = 325).

Variables	Hazard Ratio	95% Confidence Interval	*p*-Value
Age, years	1.02	1.01–1.03	<0.001
CTCs detected			<0.001
0 CTCs	reference		
1 CTCs	3.36	2.25–5.04	
2–3 CTCs	2.35	1.50–3.68	
>3 CTCs	2.86	1.58–5.17	
Total number of regional nodes with metastases			<0.001
1 LN	reference		
2–3 LN	1.64	1.19–2.27	
>3 LN	2.68	1.79–4.01	
Missing	0.80	0.45–1.42	

## Data Availability

The data presented in this study can be shared upon request.
